# Extrusion of Porous Protein-Based Polymers and Their Liquid Absorption Characteristics

**DOI:** 10.3390/polym12020459

**Published:** 2020-02-16

**Authors:** Antonio J. Capezza, Eva Robert, Malin Lundman, William R. Newson, Eva Johansson, Mikael S. Hedenqvist, Richard T. Olsson

**Affiliations:** 1 Fibre and Polymer Technology, KTH Royal Institute of Technology, Teknikringen 56, SE-100 44 Stockholm, Sweden; erobert@kth.se (E.R.); mikaelhe@kth.se (M.S.H.); 2 Department of Plant Breeding, SLU Swedish University of Agricultural Sciences, BOX 101, SE-230 53 Alnarp, Sweden; bill.newson@slu.se (W.R.N.); eva.johansson@slu.se (E.J.); 3 Essity Hygiene and Health AB, SE-405 03 Gothenburg, Sweden; malin.lundman@essity.com

**Keywords:** wheat gluten, protein, extrusion, sustainability, absorbents, porosity, circularity

## Abstract

The production of porous wheat gluten (WG) absorbent materials by means of extrusion processing is presented for the future development of sustainable superabsorbent polymers (SAPs). Different temperatures, formulations, and WG compositions were used to determine a useful protocol that provides the best combination of porosity and water swelling properties. The most optimal formulation was based on 50 wt.% WG in water that was processed at 80 °C as a mixture, which provided a porous core structure with a denser outer shell. As a green foaming agent, food-grade sodium bicarbonate was added during the processing, which allowed the formation of a more open porous material. This extruded WG material was able to swell 280% in water and, due to the open-cell structure, 28% with non-polar limonene. The results are paving the way towards production of porous bio macromolecular structures with high polar/non-polar liquid uptake, using extrusion as a solvent free and energy efficient production technique without toxic reagents.

## 1. Introduction

Porous materials from petroleum-based polymers (e.g., polystyrene and polyurethane) are widely used to produce plastic foam materials for various engineering areas [1,2,3,4]. Here, interconnected porosity is of interest and useful in fluid absorption applications relying on absorption by means of capillary action [5,6,7,8]. Absorption by means of capillary action has been of particular interest over the last few decades due to the increasing demand for daily-care products, partly related to worldwide population growth [9,10,11,12]. However, the common use of fossil-based polymers in the hygiene industry is well known and highlights the need for finding more sustainable bio-based alternative materials [11,13,14,15]. Recent studies have shown that foam structures can be effectively prepared utilizing macromolecules from nature, such as polysaccharides and/or proteins [16,17,18]. These absorbent materials have most frequently been obtained through lyophilization where the material is frozen and the water crystals being templates are sublimated in order to generate porosity [19,20]. Hemicellulose- and protein-based foams have shown a liquid absorption capacity of 12.5 and 32 g/g, respectively, demonstrating their potential to replace their petroleum-based counterparts [20,21,22]. The absorption capacities for such bio-based foams have been within the range of those for fossil-based absorbent polymers (>10 g/g) [23].

While lyophilization is the most common method for producing bio-based porous materials in the laboratory, other techniques have been reported, including more expensive super critical drying or complex Pickering emulsions using nanoparticles to stabilize the air–liquid interphase during the foam preparation [24,25,26]. The two techniques can be used to create pores with a size of the order of 20 µm and 10 nm, respectively [27,28], but, similarly with lyophilization, the techniques suffer from high cost and intense energy use in any industrial scale production [29,30]. Interestingly, it has been shown that wheat gluten (WG) proteins demonstrate such cohesive viscoelastic properties that neither of the above cost-energy expensive techniques is required for the production of porous materials with high liquid absorption (>500% in water) [31]. Gluten is therefore a particularly interesting candidate for the production of sustainable superabsorbent polymers (SAPs), not the least because WG is an agricultural industry co-product not requiring expensive and energy-demanding techniques [19,32,33,34]. Addressing the scalability issues towards production for the daily-care product market within a circular economy while taking advantage of the added value of WG as a raw material requires, however, attention to how the WG materials can be processed using large-scale foam production techniques [11,35]. Here, the most common commercial processing by far is extrusion [36,37], making it the most attractive technique for processing bio-based macromolecules and/or proteins for use as porous materials [11].

In this article, we report the extrusion of porous WG materials for the development of future scalable protocols targeting sustainable superabsorbent materials for use in daily-care product applications. Extrusion was performed on a 50 wt.% aqueous WG mixture and the most optimal conditions were established in the temperature window from 80 °C to 120 °C, resulting in extrudates with pore sizes ranging from 65 to 116 µm, composed of both open and closed pores. The open porosity, coupled with the high swelling properties of the WG, allowed a rapid liquid absorption with up to 280% weight increase in water and approximately 30% weight increase in non-polar limonene. In the developed protocol, water was used as a dispersant/blowing agent, glycerol as a processing aid, and sodium bicarbonate (NaHCO_3_) as a chemical blowing agent, which are all environmentally friendly chemicals. The results demonstrate the possibilities of using WG as a raw material for the production of porous structures with a high swelling capacity. WG is a competitive bio-based polymer that from life cycle assessment studies has shown promising environmental characteristics that are better than PLA, for example [35].

## 2. Materials and Methods 

Wheat gluten (WG) concentrate powder was provided by Lantmännen Reppe AB, Lidköping, Sweden. The WG was obtained as a product-stream from industrial wheat starch extraction with a reported protein content of 86.3 ± 0.3 (Dumas method, NMKL 6:2003, USA, nitrogen/protein conversion factor N x 6.25). The fat and ash contents of the received WG powder were 0.9% ± 0.1% and 0.8% ± 0.1%, respectively (2009/152/EU mod and NMKL 173). Glycerol (ACS reagent, >99.5%) was purchased from Sigma-Aldrich (Stockholm, Sweden). Sodium bicarbonate (ACS reagent, >99.7%) was purchased from Sigma-Aldrich (Stockholm, Sweden). The defibrinated sheep blood was provided by Disease Vector Group, Swedish University of Agricultural Sciences, Alnarp, Sweden. The extruder used was a single screw Axon AB (type BX18) having 5 heating/cooling zones along the barrel and die (4 equally distributed on the barrel and 1 on the die). The extruder was equipped with a screw having a length 375 mm and a diameter of 15 mm (L/D = 25), with a compression ratio of 2.57 (feeding zone and meeting zone depth of 3 and 1 mm, respectively).

### 2.1. Extrusion of the WG/Water Formulations

A 100 g portion of WG powder was gradually added to a beaker containing 100 mL MilliQ water (MQw), pre-adjusted to pH 11 (using 1 M NaOH). Other WG contents were also tested, but the 1:1 gluten:water mass ratio yielded the highest WG content while having good cohesive/extrusion properties. A slow addition of the WG aimed to avoid the excessive formation of granules. The pH of the mixture was continuously monitored by using pH-meter paper stripes, and corrected to 11 when needed. The pH was kept at 11 to favor WG protein denaturation and expose the protein functional groups. For the initial mixing, magnetic stirring was used, but as the mixture became too viscous (after approximately the addition of 30 g of WG), manual mixing was performed. The total mixing time for the 1:1 WG:water mix formation was approximately 15 min. Long mixing times should be avoided as it can induce alkaline crosslinking in the WG [38].

The WG mix (with solidity resembling a dough for breadmaking) was immediately transferred to the Axon AB (type BX18) extruder. The temperatures used for the extrusion were 20—50—60—70—80 °C (barrel–barrel–barrel–barrel–die) (Low Temperature profile (LT)) and 20—90—100—110—120 °C (High Temperature profile (HT)). The die was circular having a diameter of 6 mm. The screw speed was always set at 20 rpm. The extruded WG strands were placed in a forced-air oven at 40 °C for 24 h to dry the materials (post-extrusion). Thereafter, the materials were kept inside silica-containing desiccators at least 1 week prior to any test. For comparison, pieces of unextruded WG/water mix were placed into cubic molds (1 cm^3^) and then frozen at −25 °C for 24 h. Thereafter, the frozen cubes were lyophilized for 48 h and stored in the silica-containing desiccator. This sample was named WG(f). 

### 2.2. Extrusion of WG/Water/Glycerol

The sample preparation was the same as described in Section 2.1, with the only difference being that 5 wt.% of glycerol was added to the pre-adjusted pH 11 MQw before the gluten addition. This sample was also extruded using the extrusion profiles described in 2.1, that is, the LT (Low Temperature, 20—50—60—70—80 °C) and the HT (High Temperature, 20—90—100—110—120 °C) profiles. These samples were named WG 5G LT and WG 5G HT.

### 2.3. Extrusion of WG/Water/Sodium Bicarbonate 

The sample preparation was the same as described in Section 2.1. However, after the mixing of WG with the water pre-adjusted to pH 11, 5 g of NaHCO_3_/100 g WG were added to the mixture and thoroughly mixed before the extrusion. These samples were named WG 5S LT and WG 5S HT.

### 2.4. Extrusion of WG/Water/Glycerol/Sodium Bicarbonate 

The sample preparation was the same as described in Section 2.2. However, after the mixing of WG with water, pre-adjusted to pH 11 (containing 5 wt.% of glycerol), 5 g of NaHCO_3_/100 g WG were added to the dough and thoroughly mixed before the extrusion. These samples were named WG 5G5S LT and WG 5G5S HT. A summary of the different sample names and formulations is described in Table 1.

### 2.5. Swelling

The swelling capacity (SC) of the samples was determined by completely immersing pieces of the samples in MQw or limonene. The immersion times were 10, 30, and 1440 min (24 h). After removing the samples from the liquid, they were placed on a tissue paper for 10 s to remove the excess liquid. The swelling was calculated according to Equation (1), and the results are reported as averages of triplicates, with standard deviations. Parts of some dried extruded WG samples were ground in a mortar until a powder was obtained. This was made to assess the free liquid swelling of the samples (free swelling capacity (FSC)) and to eliminate the effect of having closed cells in the materials. The FSC was calculated using the Nonwovens Standard Procedure (NWSP 240.0.R2) “tea-bag” [39,40]. Briefly, approximately 460 mg of extruded dry materials was added to a nonwoven fabric bag having a dimension of 40 × 60 mm^2^ (mesh = 400). The bag containing the material was hooked on a rod and immersed in a beaker containing MQw. The immersion time was the same as before. After the immersion, the bag was hung for 10 s outside the liquid and then placed on a tissue paper for 10 s to remove unabsorbed water. Three empty bags were handled identically to obtain an average correction factor (W_b_) using Equation (2). The FSC was estimated using Equation (3) and the results are reported as the average of triplicates.

(1) $$\mathrm{SC}=(W_{2}-W_{1})/W_{1}$$

(2) $$W_{b}=W_{s}/W$$

(3) $$\mathrm{FSC}=\left(\left[W_{i}-\left(W_{0}\times W_{b}\right)\right]-W_{d}\right)/W_{d}$$

W_1_ is the dry weight of the extruded specimen, and W_2_ is the swollen weight of the specimen. W_s_ and W are the weights of the wet and dry blank bags, respectively. W_i_ is the weight of the swollen material, W_0_ the weight of the dry bag used, and W_d_ the weight of the added dry ground sample. 

An additional absorption test using defibrinated sheep blood was performed on some samples [39]. The sheep blood absorption was used to show the swelling capacity of other liquids important for the daily-care product industry. The immersion time was always 30 min and the swelling was calculated using Equation (1), and the results are reported as the average of duplicates.

### 2.6. Density Measurements 

The density was calculated using two methods. The apparent density ($\rho_{a}$) was calculated using the weight of the dry samples (using a Mettler Toledo AL104 balance) divided by its volume (assuming a cylindrical shape), and the Archimedes principle.

The weights of the samples were measured in air and in n-heptane, and the Archimedes density (ρ) was calculated using Equation (4) as follows:

(4) $$\rho =\left(A/\left(A-B\right)\right)\cdots \left(\rho_{o}-\rho_{1}\right)+\rho_{1}$$

where A and B are the weight of the sample in air and n-heptane, respectively, $\rho_{o}$ is the density of n-heptane (0.6838 g/cm^3^), and $\rho_{1}$ is the density of air (0.0012 g/cm^3^). The porosity was estimated by using Equation (5) and the apparent density of each material ($\rho_{a}$) and taking the raw WG powder density as 1300 kg/m^3^ [31]. The closed cell porosity was estimated by means of Equation (6), using the density of the raw WG powder (1278 ± 49 kg/m^3^) and the density of each sample (ρ) calculated from the Archimedes method (Equation (4)). The lower density of the WG powder obtained by the Archimedes method is a consequence of the method not considering air trapped in the particles.

(5) $$\mathrm{Porosity}\;\left(\%\right)=\left[1-\left(\rho_{a}/1300\right)\right]\cdots 100$$

(6) Closed cell porosity (%)=[1−(ρ/1278)]⋯100

### 2.7. Fourier-Transform Infrared Spectroscopy (FTIR)

The effect of the extrusion processing on the WG was studied using FTIR. The FTIR spectrum was obtained using a PerkinElmer Spectrum 100 coupled to a Golden Gate unit (Single-reflection ATR, Graseby Specac Ltd, Kent, England). A triglycine sulphate (TGS) detector was used with a resolution of 4.0 cm^−1^, and 32 consecutive scans were performed per sample. The samples were dried in a desiccator over silica for 1 week before the runs. A peak deconvolution was performed as reported by Cho et al. [41] using the PerkinElmer Spectrum software (version 10.5.1 (2015)) with an enhancement factor (γ) of 2 and a smoothing filter of 70%.

### 2.8. Scanning Electron Microscopy (SEM)

The morphology of the different samples was investigated using a Hitachi TM-1000 tabletop SEM (Tokyo, Japan) (10 kV voltage, 6 mm working distance, backscattering mode). The extruded samples were immersed in liquid nitrogen for 5 min and then fractured. The cryo-fractured cross-sections of the samples were placed on a conductive carbon tape. To evaluate the structure of the samples after being immersed in MQw for 24 h, the extruded swollen samples were frozen at −25 °C during 24 h and then lyophilized for 48 h. The lyophilized samples were immersed in liquid nitrogen and cryo-fractured. The surface morphology was analyzed using a Hitachi S-4800 field emission scanning electron microscope (FE-SEM). A voltage of 3 kV and a current of 10 µA were used. The lyophilized materials were sputtered with a palladium/platinum (Pt/Pd) target in an Agar High Resolution Sputter Coater (model 208RH). The sputtering time for all samples was 45 s proving an estimated conductive layer of 1–2 nm. The pore size of the extrudates was estimated using ImageJ^®^ [42], taking at least 50 measurements and reporting the average and standard deviation. The pore size measurements were made using 500× images, and when no porosity was observed at this magnification, the sample was considered non-porous. 

### 2.9. Thermal Gravimetric Analysis (TGA)

The thermal stability of the extruded WG samples was studied using a Mettler-Toledo TGA/SDTA851 instrument (Leicester, England). An amount of 5.0 ± 0.03 mg of the ground material was put in 70 μL alumina crucibles. The method consisted of a drying step of 10 min at 50 °C followed by heating from 50 °C to 800 °C (10 °C/min). A nitrogen flow of 50 mL/min was used during the tests. 

## 3. Results and Discussion

### 3.1. Physical Characteristics 

**Figure 1:**
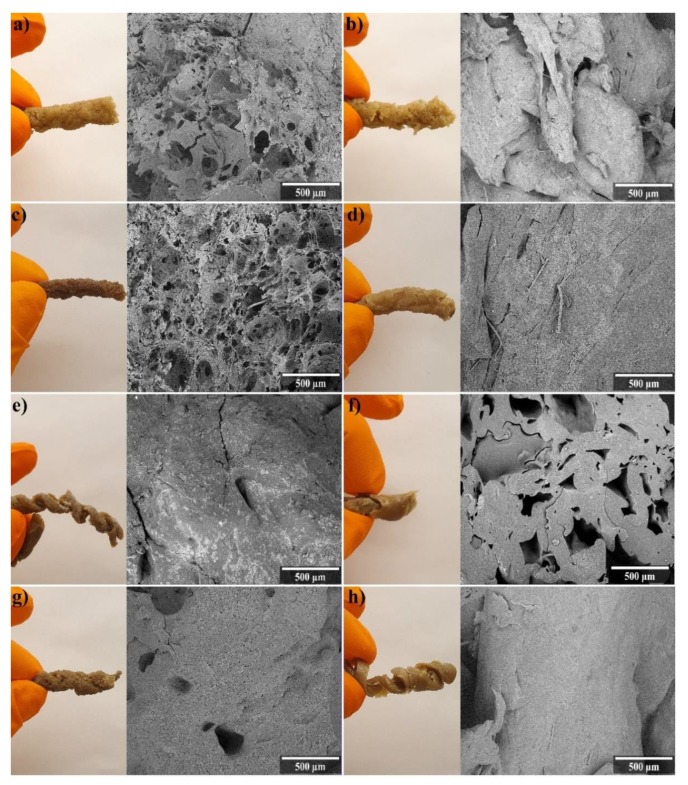
Images of the WG extrudates after drying and their respective SEM cross-sections. The panels show (**a**) WG LT, (**b**) WG HT, (**c**) WG 5G LT, (**d**) WG 5G HT, (**e**) WG 5S LT, (**f**) WG 5S HT, (**g**) WG 5G5S LT, and (**h**) WG 5G5S HT.

Figure 1a shows that the extrusion of WG at low temperature (WG LT) provided a porous structure inside the sample and a denser structure as the outer shell. The same porous characteristics were observed for the WG sample containing 5 wt.% glycerol, also extruded at low temperature (WG 5G LT), Figure 1c. Both WG LT and WG 5G LT could be extruded with essentially a cylindrical shape of the extrudate and a pore size of 85 µm and 65 µm, respectively. The diameter of the inner porous core of the WG LT sample was approximately 2 mm, whereas it was 2.5 mm for WG 5G LT. Glycerol, a known protein plasticizer and a processing aid, made the WG more stretchable and allowed more vapor-generated expansion at the exit of the die before the gases exited the extrudate surface. However, when these samples were extruded at higher temperatures (here, 120 °C at the metering zone), the porosity in the core, previously obtained at 80 °C, was absent (see Figure 1b,d). The cylinder-like shape was also obtained for the WG HT and WG 5G HT samples, but a temperature above 100 °C was previously reported to lead to extensive crosslinking with disulphide (–S–S–) bonds in WG materials. Therefore, the high temperature and crosslinked WG matrix when extruded at high temperature could hinder the expansion generated by the water vapor in the WG extrudate when exiting the extruder. This could also explain the absence of the inner porous core in WG HT and WG 5G HT. In addition, the HT generated shorter and more discontinuous products due to pressure variations at the extruder die, thereby affecting the production rate for this protocol (see Table 1).

Figure 1e shows that the addition of sodium bicarbonate (NaHCO_3_) to the WG aqueous mixture (here, WG 5S LT) did not lead to any porosity at low extrusion temperatures. In addition, the cylindrical shape was lost (Figure 1e), and a helical shape originally from the flow inside the extruder barrel was obtained. The effect is a consequence of not having a “breaker plate” or a coarse screen between the barrel and the die. The high magnification images of WG 5S LT revealed the presence of aggregates, which are likely WG aggregates formed as the result of protein tendons stretched across the bubbles before they collapsed and then relaxed into spheres (Figure S1a). However, contrary to WG HT and WG 5G HT, the WG 5S HT sample showed numerous irregular open pores of approximately 300 µm in size (Figure 1f). When observed at higher magnifications, the WG 5S HT sample had a structure that suggests that the micropores collapsed when exiting the extruder (Figure S1b). The now formed irregular open pores in WG 5S HT can be the result of the effective decomposition of the NaHCO_3_ forming CO_2_ (g). The CO_2_ (g) formation from NaHCO_3_ is indeed more favored at temperatures above 115 °C [43], explaining the increased pore expansion obtained for WG 5S HT compared to WG 5S LT. 

The combination of glycerol and NaHCO_3_ in samples extruded at low temperatures (WG 5G5S LT, Figure 1g) resulted in irregular cylindrically shaped extrudates with occasionally observed 300 µm large pores. The high magnification of this sample reveals a similar collapsed porous structure as that for WG 5S HT, but with the presence of several WG aggregates (Figure S2a) similar to the ones observed on the WG 5S LT sample (Figure 1e). The WG 5G5S HT sample (Figure 1h) had a helical shape similar to WG 5S LT, and a dense structure even on a microscale (Figure S2b). Therefore, the combination of glycerol and NaHCO_3_ favored the formation of a more regular extrudate (compare WG 5S LT and WG 5G5S LT, Figure 1), while at high temperatures the structure obtained was dense. The more regular inner core porosity and cylindrical shape obtained for WG 5G LT suggest that the addition of only glycerol provides a greater water vapor-generated expansion at the extruder die. Likewise, when an open porosity is desired, the addition of NaHCO_3_ and the use of a high temperature is the best combination. The results, however, pave the way for future work on glycerol/NaHCO_3_ mass ratio optimization and the use of new bio-based foaming agents in order to optimize the formation of more regular and continuous extrudates having mainly an open-pore structure. 

Using the apparent density of the cylinders, it was estimated that the samples WG LT and WG 5G LT had a porosity of 36% and 16%, respectively (Table 2). Due to the irregular shape of some samples, the apparent density was not calculated (not measured (NM), Table 2). The above-mentioned samples also resulted in the presence of closed pores (i.e., 7% for WG LT and 3% for WG 5G LT), according to the Archimedes density results. This agrees with the SEM images, showing a porous core and a denser skin layer in these samples (Figure 1a–c). However, the WG 5S HT sample resulted in the highest closed-pore content (12%, Table 2). This shows that not all the large pores observed in the SEM micrographs were accessible for the liquid penetration (Figure 1f). The porosity of WG LT shows that by the use of only water together with the WG in the extrusion, approximately 48% of the porosity of the lyophilized foam (WG(f)) can be obtained (Table 2). Moreover, when sodium bicarbonate was used in the formulations (WG 5S HT), the porosity reached 53%, representing 73% of the porosity of WG(f) (Table 2). Although the pore size of the WG 5S HT sample was approximately four times larger than that of WG(f), the results show the potential of using NaHCO_3_ as foaming agent in the gluten formulation in combination with high temperatures. The core porosity obtained in WG 5S HT (i.e., using the continuous extrusion process) also represents approximately 50% of the porosity obtained in our previous work by the utilization of a non-continuous technique to produce WG porous structures [31]. Briefly, WG was mixed with water and glutaraldehyde, then the water was decanted from the mixture and the porous material was dried for 24 h [31]. At high magnifications (Figures S1 and S2) of WG 5S LT, WG 5S HT, and WG 5G5S LT, a microporosity with a size of 3 ± 2 µm, 7 ± 4 µm, and 4 ± 3 µm, respectively, was observed. All samples had high pore size variation (both large and small pores were considered for the calculations, Figure 1 and Table 2). Generally, the closed-pore fraction was a low percentage of the total volume, being the highest for WG 5S HT (12%) and lowest for WG 5G HT (1%).

### 3.2. Swelling

**Figure 2:**
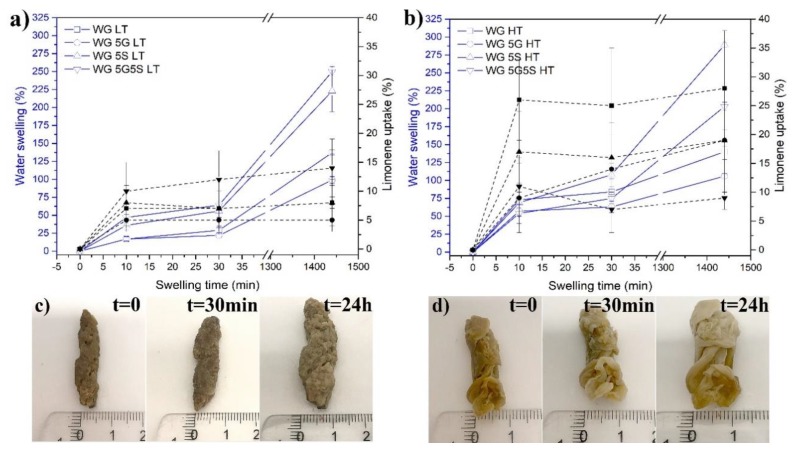
Liquid uptake in the different samples at low (**a**) and high (**b**) extrusion temperatures. A representative specimen of WG 5G5S LT (**c**) and WG 5S HT (**d**) after a 24 h swelling in water are shown. The continuous line with hollow symbols corresponds to water swelling, and the dashed lines with filled symbols correspond to limonene uptake.

All samples showed water swelling scenarios demonstrating the following characteristics; reaching a first plateau within 10–12 min, which remained stable for approximately 20 min, followed by an increase after 30 min (Figure 2a). This behavior was previously observed for WG-superabsorbent materials and was due to the plasticization effect of water on the gluten protein [21]. The highest water swelling was obtained for WG 5S HT after 30 min and 24 h, 100% and 290%, respectively (Figure 2b). This was due to the large open pores observed in the sample (Figure 1f), which allowed the water to spread out easily and interact with a greater WG surface. Note that for the same sample at low extrusion temperatures (WG 5S LT), the water swelling was 225%, also demonstrating the effect of the large pores. With 250% water uptake (24 h, Figure 2a), the WG 5G5S LT sample showed the second largest water swelling. This result was again related to the large pores observed in the sample, together with several collapsed micropores present (Figure S2). The large swelling ratios are illustrated by the increase in volume of the previous samples, WG 5G5S LT (Figure 2c) and WG 5S HT (Figure 2d). The short time (30 min) and long time (24 h) water swelling are important swelling times for sanitary and other applications such as agriculture, respectively. The WG 5S HT sample was stable and showed a sponge-like behavior even after 24 h, and could sustain repeated manual compression for removing the water (Video S1). Thus, a sponge/elastic behavior similar to the one previously obtained with lyophilized foams can also be obtained using the extrusion technique [20,21,31]. However, when the WG 5G5S LT sample was compressed, the material did not maintain its shape to a 100% degree (Video S1). Previously, we showed that non-lyophilized WG porous materials using glutaraldehyde as protein network stabilizer were able to swell 468% in water [31]. The values reported here show approximately 50% of the maximum swelling of the previously reported values using glutaraldehyde. Although the maximum swelling ratios are lower, the combination of the commercially attractive extrusion technique and the absence of toxic crosslinking agents represents a promising continuation of studies for the production of environmentally friendly absorbent materials.

Limonene was used to evaluate the non-polar liquid uptake capacity of the different samples, and all samples reached the maximum limonene uptake after 10 min. The capillary action responsible for this rapid uptake is an important parameter for non-polar liquid superabsorbent applications. The highest limonene uptake was achieved in the WG HT sample (30%). WG 5G LT and WG LT showed the lowest limonene uptake (5% and 8%, respectively). The reason for the low limonene uptake for the samples was likely due to the dense skin layer observed (Figure 1a,c), which hindered and slowed the limonene (and also water) penetration into the inner part of the material. For an illustration of the rapid limonene uptake, see Video S2 where the WG HT sample was placed in contact with limonene. 

**Figure 3:**
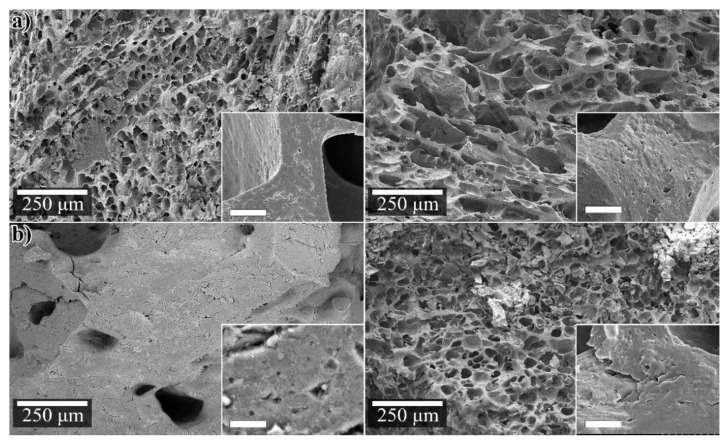
(**a**) WG(f) (left), WG(f) after 24h swelling (right); (**b**) WG 5G5S LT (left), after 24h swelling and lyophilized (right). The bar in the insert indicates 5 µm.

The lyophilization of the WG 5G5S LT sample after being immersed 24 h in water revealed that the large scattered pores and the more or less solid pore walls were absent. Instead, a highly porous structure was obtained (pore size of 34 ± 10 µm), which could be ascribed to the sublimation of the water crystals formed from freezing of the originally highly swollen cylinder. In fact, the porous microstructure resembled the structure of the originally freeze-dried reference WG foam (WG(f)) (Figure 3a), having a pore size of 27 µm (Table 2). Figure 3a shows that the WG(f) foam kept approximately the same microstructure even after a second lyophilization (after being swollen in water for 24 h, Figure 2a right). Compared to the original WG(f) foam, the WG(f) material freeze-dried twice had thicker cell walls (>15 µm) and a microporosity within the cell walls. The pores in WG(f) after the second lyophilization were slightly larger than in the original foam (Table 2), here 75 ± 46 µm. The newly developed porosity in WG 5G5S LT also illustrates that the water penetrated the entire cross-section of the sample homogenously (Figure 3b). 

**Figure 4:**
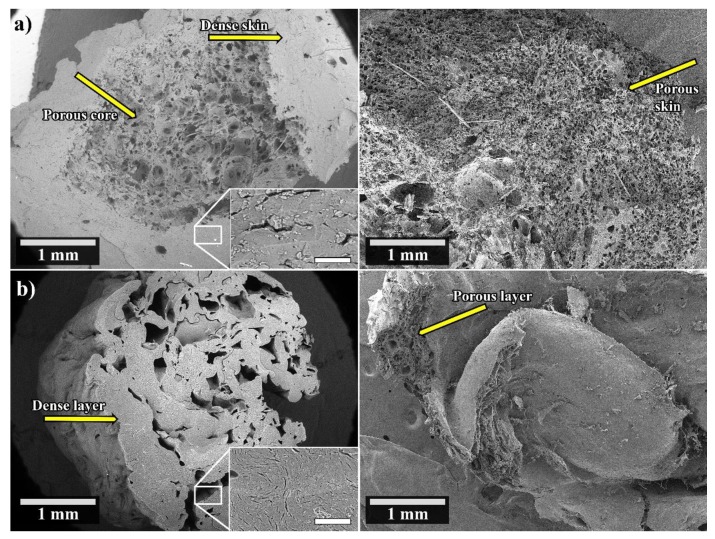
Cross-section of the (**a**) WG 5G LT (left) and WG 5G LT after 24 h swelling and lyophilized (right); (**b**) WG 5S HT (left) and WG 5S HT after 24 h swelling and lyophilized (right). The bar in the insert indicates 50 µm.

As previously discussed, the WG 5G LT sample consisted of a porous core structure with a denser skin, whereas the WG 5S HT cross-section revealed a dense layer having large pores that had collapsed (Figure 4a, Figure 4b, respectively). To demonstrate the effect of the water penetration within the dense layers of the latter samples after 24 water swelling, the lyophilized cross-sections of WG 5G LT and WG 5G LT are shown in Figure 4. The dense skin obtained in the extruded WG 5G LT (Figure 4a, left) changed to a highly porous skin (Figure 4a, right). The formed porous skin showed a homogenous pore size of 30 ± 12 µm with an increase in the cross-section from approximately 0.9 mm to 2 mm after 24 h swelling, as a consequence of the volumetric expansion due to water swelling in the dense skin. However, the porous core observed in WG 5G LT before the water swelling (Figure 4a, left) did not show a visible change after the swelling and lyophilization (Figure 4b, right). This indicated that water did not swell the cell walls of the porous core in the sample. Likewise, the dense layer observed for the WG 5S HT sample also changed to a porous layer after the swelling and lyophilization of the sample (Figure 4b, right). To further demonstrate the effect of the dense skin in the water penetration, the WG 5G LT sample was ground to a powder and the tea-bag test was performed. The WG 5G LT powder sample had a 10 min, 30 min and 24 h water free swelling capacity (FSC) of 184% ± 30%, 217% ± 28%, and 237% ± 9%, respectively. These values represent an increase of the short (10 min) and long-term (24 h) water swelling of approximately 995% and 140%, respectively, compared to the non-ground WG 5G LT sample.

**Figure 5:**
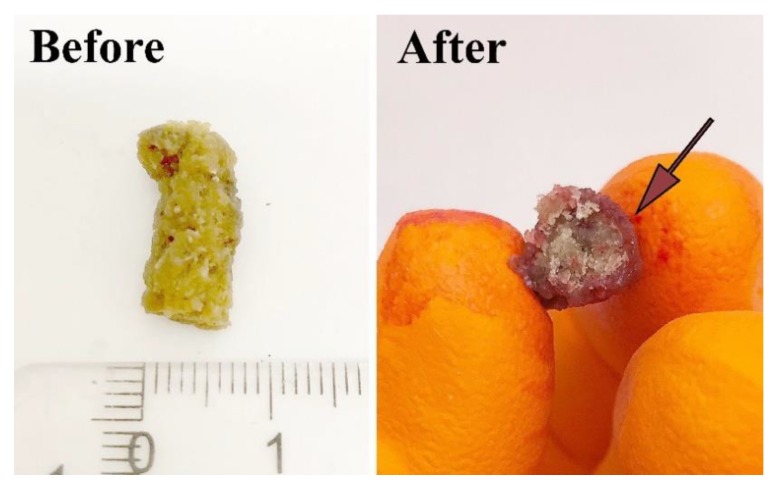
WG 5G5S LT before immersion in defibrinated sheep blood and a cut of the cross-section after 30 min immersion in the liquid.

Defibrinated sheep blood absorption (30 min) in the WG 5G LT, WG 5S HT, and WG 5G5S LT samples showed an uptake capacity of 22.1% ± 0.2%, 35.7% ± 6.7%, and 36.3% ± 1.6%, respectively. The increased blood uptake of WG 5G5S LT compared to WG 5G LT, the first having an irregular large pore structure (Figure 3b), demonstrated that the effects of the capillarity action seen in WG 5G5S LT were reduced in WG 5G LT due to its dense skin structural feature (Figure 4a). Figure 5 shows the WG 5G5S LT sample before and after the 30 min defibrinated sheep blood uptake test. The blood had penetrated only parts of the outer portion of the sample, and essentially not the core of the sample, which thereby decreased the blood swelling capacity of the sample. However, the considerable increase in the water swelling capacity obtained when the WG 5G LT sample was ground to powder suggests that grinding could be used as a beneficial post-process for increasing the liquid swelling of the WG extrudates and favoring the water/liquid spreading throughout the entire sample.

### 3.3. Protein Thermal and Structural Characteristics

**Figure 6:**
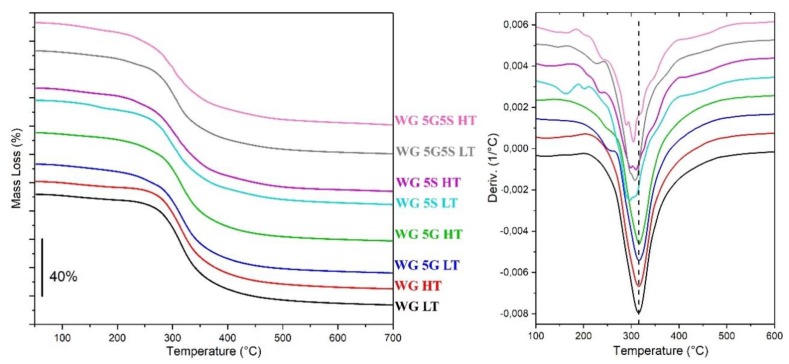
Thermal stability of the samples (left) and first derivative (right). The curves were stacked for clarity.

Figure 6 shows the thermal stability of the different samples produced. The onset temperature of degradation and the maximum mass loss rate of each sample are summarized in Table 3, including the results for the TGA analysis of the as-received WG powder. The samples WG LT, WG HT, WG 5G LT, and WG 5G HT showed no significant difference in the thermal stability with respect to the onset of weight loss and the maximum mass loss rate (Table 3). However, the increase in approximately 15 °C in the maximum mass loss rate for the above-mentioned samples compared to the raw WG indicated the effect on the gluten by the thermal/alkali treatment when extruded, possibly inducing crosslinking in the protein and thereby increasing their thermal properties [38,44,45]. On the other hand, all the samples treated with sodium bicarbonate, namely, WG 5S LT, WG 5S HT, WG 5G5S LT and WG 5G5S HT, had a lower onset and maximum mass loss rate temperatures than the other extruded samples (Table 3). The decrease in the thermal stability of the samples where sodium bicarbonate was used suggests that the NaHCO_3_ could also influence the group functionality of the WG protein. This is in accordance with previous reports, where the functionalization of proteins was shown to influence the thermal behavior of the protein samples [46,47]. Additionally, the modification of the functional/charged groups in the WG can contribute to the increase in water swelling due to charge repulsion in the material [11], which is in line with the higher water swelling results obtained for WG 5G5S LT and WG 5S HT, for example (approximately 280%, see Figure 2). The TGA profiles of the neat sodium bicarbonate (Figure S3) show that at 120 °C the bicarbonate starts to degrade, forming CO_2_ (g). This again corresponds with the large porous structure obtained for WG 5S HT, as discussed previously in Section 3.1. The results also suggest the potential for future work related to the method herein suggested utilizing higher extrusion temperature for an even larger foaming effect of the NaHCO_3_ through its thermal degradation. 

**Figure 7:**
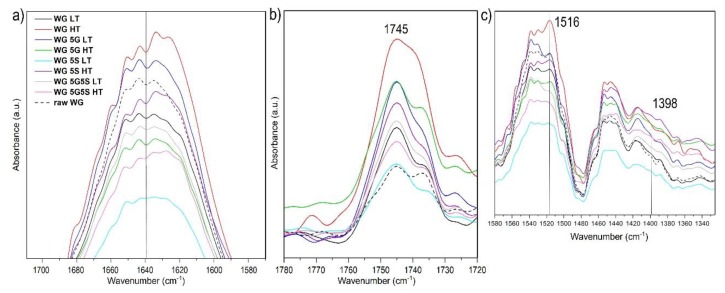
FTIR of the amide I region (**a**), the carbonyl region (**b**) and 1550–1340 cm^−1^ region (**c**) for the different samples.

The FTIR amide I region of the extruded WG materials, as well as the as-received WG powder, reflects the protein secondary structure, as seen in Figure 7a. The full FTIR spectra of all samples are shown in Figure S4. Overall, all the extruded samples (with the exception of WG 5S LT) showed a change/shifting in the amide I region (1680–1580 cm^−1^) compared to the neat WG powder, indicating an increase in the content of β-sheet structure and decreased α-helix content for the extruded samples. The largest shift and strongest peaks at lower wave numbers (below 1635 cm^−1^) corresponding to β-sheets was observed for the WG HT sample, whereas a smaller shift was apparent for WG 5S LT. High temperatures are known to favor WG protein aggregation/crosslinking [48], which then influences protein thermal stability and increases the β-sheet content, as reflected in the amide I spectra (Figure 7a). On the other hand, the samples containing glycerol (both at low and high temperatures) did not display major changes in the secondary structure when compared to WG HT and WG 5S HT (Figure 7a). This agrees with the fact that glycerol acts as a protein plasticizer, increasing the mobility of the chains and reducing their inter/intra-molecular interactions [49,50].

An increase in the carbonyl region intensity (1760–1720 cm^−1^) for all samples compared to the neat WG (except for WG 5S LT) suggests that the protein carbonyl functionality was exposed after the temperature/alkali treatments (Figure 7b). The shoulder observed at 1398 cm^−1^ for WG 5S HT suggests that there is an increase in the carbonyl functionality in the protein coming from the formation of sodium-neutralized carboxylic acid groups (symmetric stretching) [51,52]. The results agree with the one showing that the highest water swelling was observed for the WG 5S HT sample (coupled with the large open-cell structure), thereby indicating an apparent partial functionalization of the gluten macromolecules by the sodium bicarbonate. Sodium bicarbonate as a simultaneous foaming and functionalization agent of WG materials is thus suggested as an interesting reagent for the production of extruded protein-based materials [11,14]. The functionalization of protein side-streams, such as wheat gluten (WG), has been shown to be beneficial in increasing their water swelling properties, making them more attractive as compared to the current non-sustainable synthetic SAPs [40].

## 4. Conclusions

Extruded porous wheat gluten (WG) with water, glycerol, and sodium bicarbonate as green reactants was evaluated. The wheat gluten extruded with sodium bicarbonate at 120 °C showed a porous structure that allowed >275% water uptake while having highly resilient properties when repeatedly compressed. The interconnected pores also allowed the WG material to take up >20% of a non-polar liquid (limonene). Extrusion of only WG and water (1:1 ratio) at low temperature (80 °C) resulted in extruded materials having a porosity of approximately 36%, which was located in the core of the extrudate. Thus, it was demonstrated that using the common polymer processing technique of extrusion can be foreseen as a potential technique to produce porous WG protein materials with a high swelling capacity. The results pave the way for the continuous production of sustainable and biodegradable polymers with high liquid uptake, which currently are represented by synthetic materials in the daily-care product industry.

## Figures and Tables

**Table 1:** Sample formulations and the extrusion parameters used. ^1^

Sample Name	WG (wt.%)	Water (wt.%)	Glycerol (wt.%)	Sodium Bicarbonate (g/100g WG)
WG LT	50	50		
WG HT	50	50		
WG 5G LT	50	45	5	
WG 5G HT	50	45	5	
WG 5S LT	50	50		5
WG 5S HT	50	50		5
WG 5G5S LT	50	45	5	5
WG 5G5S HT	50	45	5	5

^1^ LT: low temperature: 20—50—60—70—80 °C (± 10 °C), HT: high temperature: 20—90—100—110—120 °C (± 10 °C). All the samples were extruded at a screw speed of 20 rpm. The production rate was 6.6 g/min (0.4 kg/h) and 3.24 g/min (0.2 kg/h) for LT and HT, respectively.

**Table 2:** Physical properties of the different samples.

Sample Name	Apparent Density (kg/m^3^)	Archimedes Density (kg/m^3^)	Porosity with Density (%)	Closed Cells (%)	Pore Size (µm) ^1^
WG(f)	351 ± 29	NM	73	NM	27 ± 13
WG LT	835 ± 89	1193 ± 12	36	7	85 ± 67
WG HT	NM	1287 ± 9	NM	0	NP
WG 5G LT	1093 ± 140	1246 ± 3	16	3	65 ± 50
WG 5G HT	665 ± 68	1263 ± 15	49	1	NP
WG 5S LT	NM	1280 ± 18	NM	0	NP
WG 5S HT	612 ± 83	1150 ± 34	53	12	116 ± 138
WG 5G5S LT	NM	1223 ± 25	NM	4	NP^2^
WG 5G5S HT	NM	1293 ± 8	NM	0	NP

The density of the raw WG powder according to Archimedes was 1278 ± 49 kg/m^3^ and the reported density was 1300 kg/m^3^. NM: not measured. ^1^ The pore size was measured on the 500× SEM images. ^2^ If no porosity was possible to observe at this magnification, it was considered as a non-porous sample (NP).

**Table 3:** Summary of the thermal properties obtained from the TGA curves.

Sample	Onset Temp. (°C)	Max. Mass Loss Rate (°C)
Raw WG	274	302
WG LT	278	315
WG HT	278	316
WG 5G LT	276	316
WG 5G HT	273	317
WG 5S LT	269	298
WG 5S HT	259	311
WG 5G5S LT	267	307
WG 5G5S HT	259	305

## Supplementary Materials

The following are available online at https://www.mdpi.com/2073-4360/12/2/459/s1, Figure S1: SEM micrographs of WG 5S LT (a) and WG 5S HT (b); Figure S2: SEM micrographs of WG 5G5S LT (a) and WG 5G5S HT (b); Figure S3: TGA and first derivate (inset) profiles of the sodium bicarbonate; Figure S4: Full FTIR spectra of all the samples extruded, including the as-received WG powder (raw WG); Video S1: Sponge-like effect of WG 5S HT and WG 5G5S LT after water swelling; Video S2: Rapid limonene uptake of the extruded WG samples.
