# Supplementary material for: Extrusion of Porous Protein-Based Polymers and Their Liquid Absorption Characteristics
Source: Polymers (Basel). 2020 Feb 16;12(2):459. doi: 10.3390/polym12020459 (PMC7077648; doi:10.3390/polym12020459)
Supplement: Supplementary file 1 [file polymers-12-00459-s001.zip › Supplementary Info ERobert V2.docx]

**Supporting information**

Extrusion of Porous Protein-Based Polymers and Their Liquid Absorption Characteristics

*Antonio J. Capezza^1,2*^, Eva Robert^1^, Malin Lundman^3^, William Newson^2^, Eva Johansson^2^, Mikael S. Hedenqvist^1^, Richard T. Olsson^1*^*

^1^Fibre and Polymer Technology, KTH Royal Institute of Technology, Teknikringen 56, SE-100 44 Stockholm, Sweden.

^2^Department of Plant Breeding, SLU Swedish University of Agricultural Sciences, BOX 101, SE-230 53 Alnarp, Sweden.

^3^Essity Hygiene and Health AB, SE-405 03; Gothenburg, Sweden, malin.lundman@essity.com.

*Correspondence: ajcv@kth.se; Tel.: +46-762-301654 (A.C.), rols@kth.se; Tel.: +46-732-701868 (R.O.)

Number of pages: 4

Number of figures: 4


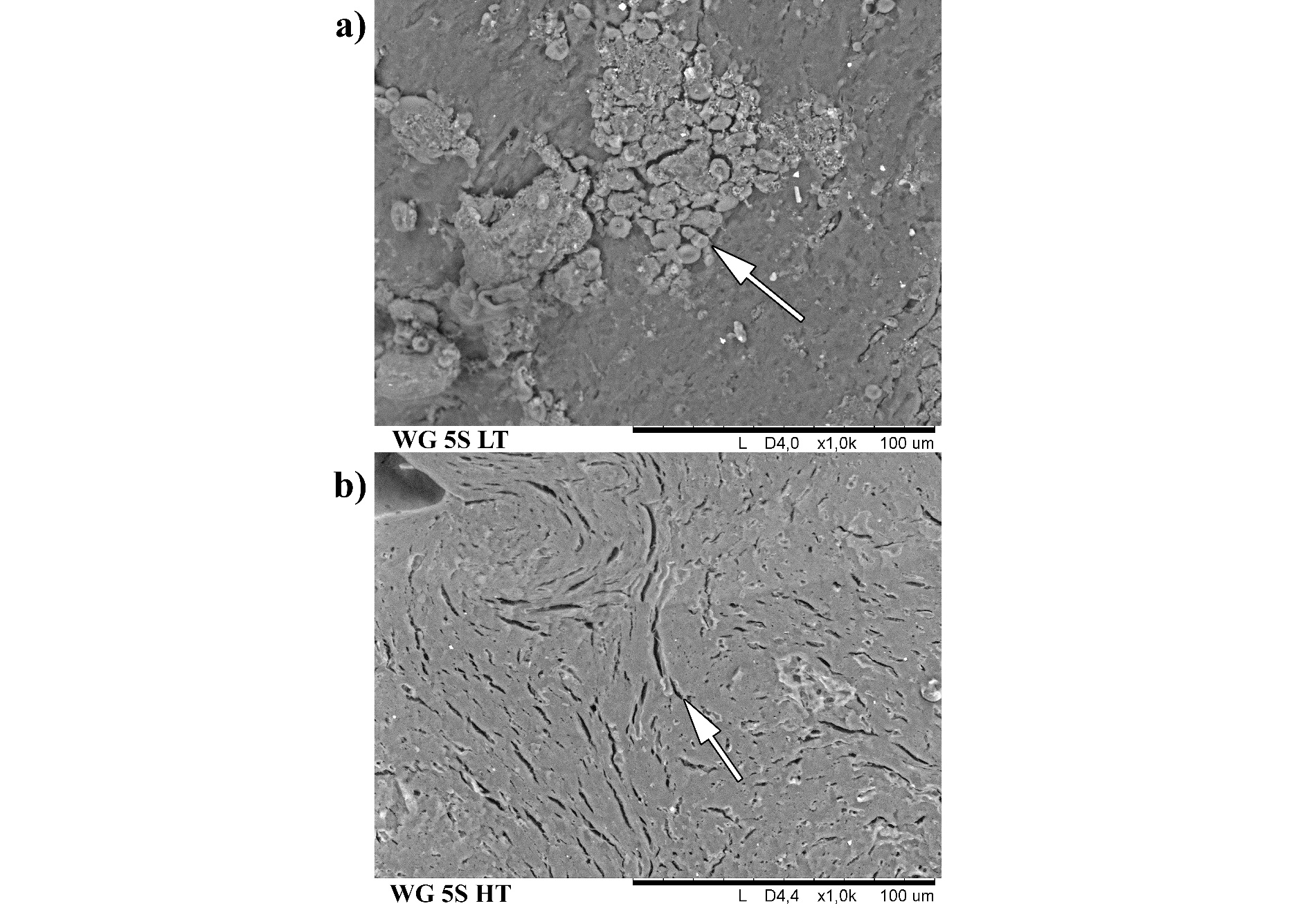


**Figure S1.** SEM micrographs of WG 5S LT (a) and WG 5S HT (b). The arrows point the WG aggregates and the collapse porous structure, a and b, respectively.


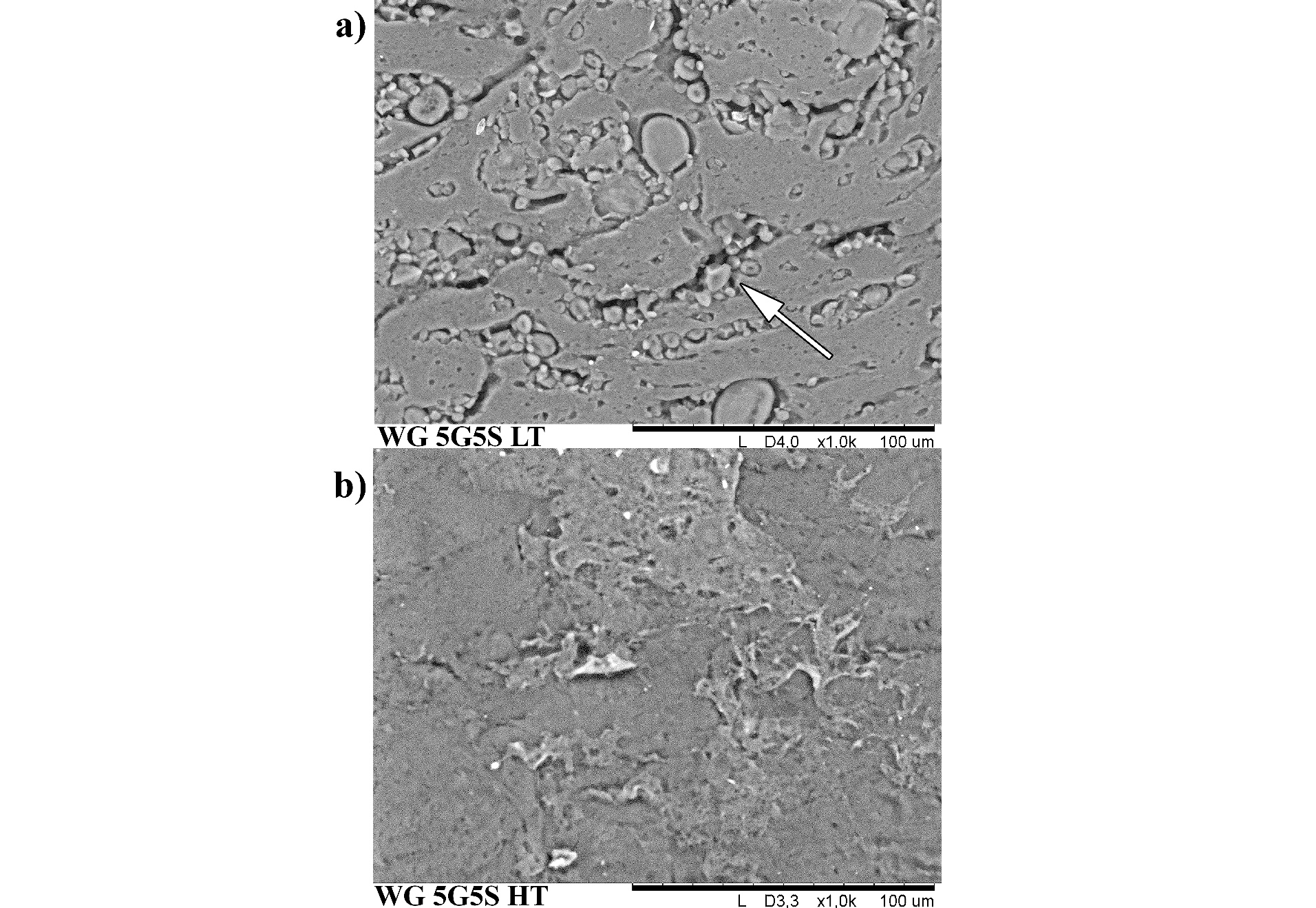


**Figure S2**. SEM micrographs of WG 5G5S LT (a) and WG 5G5S HT (b). The arrow in “a” points the WG aggregates embedded in the collapse porous structure.


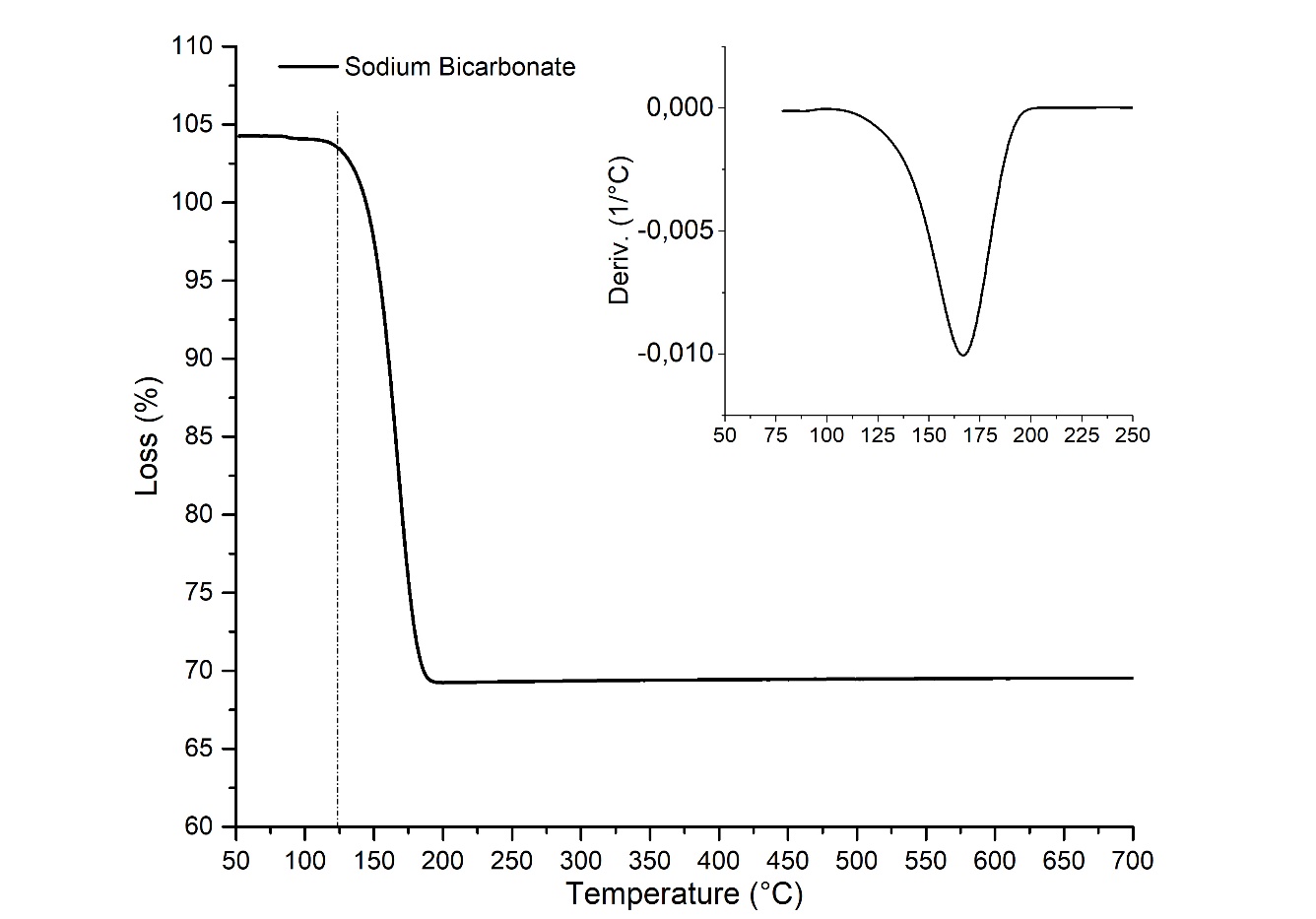


**Figure S3**. TGA and first derivate (inset) profiles of the sodium bicarbonate.


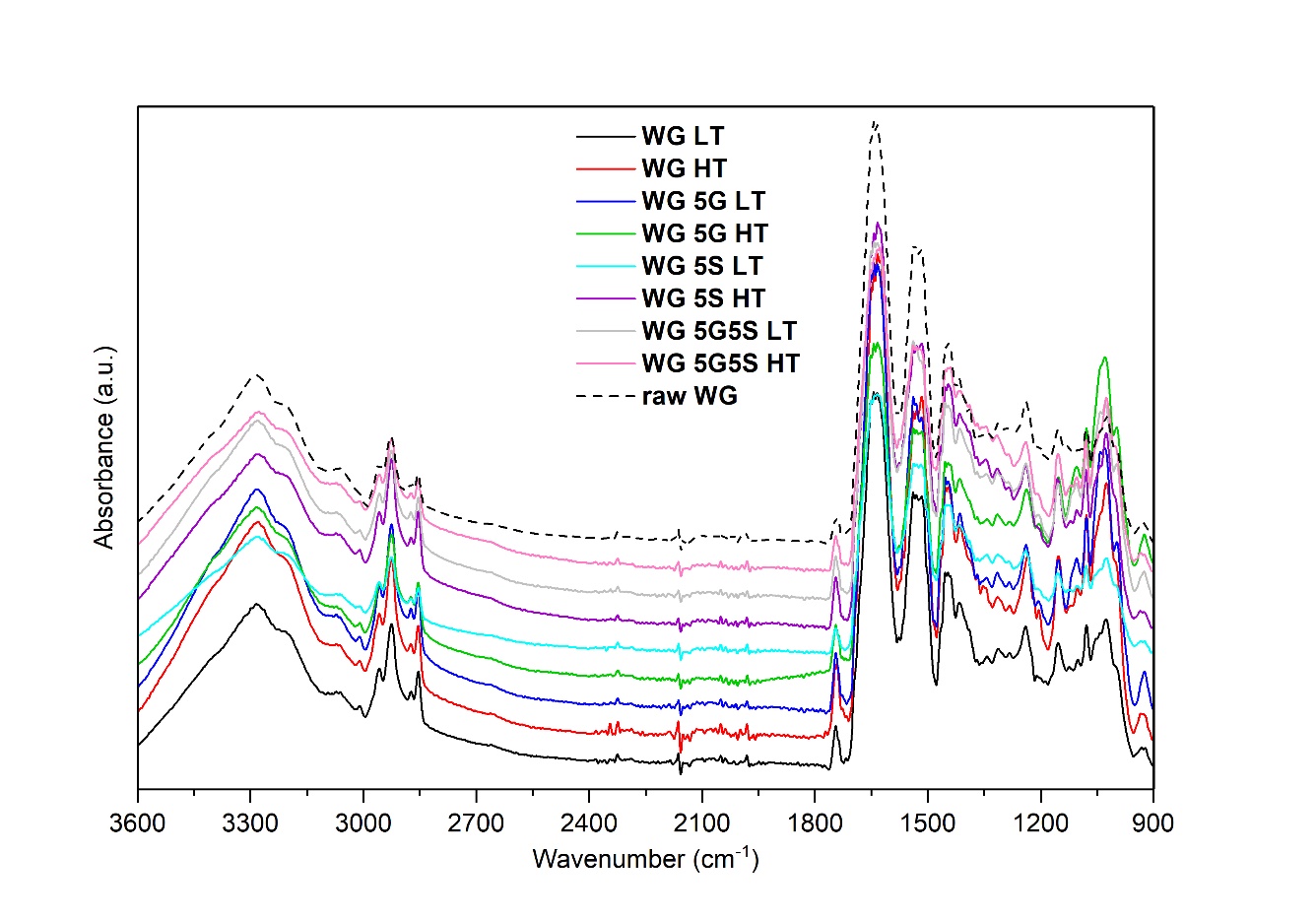


**Figure S4**. Full FTIR spectra of all the samples extruded, including the as received WG powder (raw WG).
